# Linezolid Exerts Greater Bacterial Clearance but No Modification of Host Lung Gene Expression Profiling: A Mouse MRSA Pneumonia Model

**DOI:** 10.1371/journal.pone.0067994

**Published:** 2013-06-27

**Authors:** Jiwang Chen, Gang Feng, Yang Song, Juliane B. Wardenburg, Simon Lin, Ichiro Inoshima, Michael Otto, Richard G. Wunderink

**Affiliations:** 1 Department of Medicine, Northwestern University, Chicago, Illinois, United States of America; 2 Section of Pulmonary, Critical Care Medicine, Allergy and Sleep, University of Illinois at Chicago, Chicago, Illinois, United States of America; 3 Northwestern University Biomedical Informatics Center, Chicago, Illinois, United States of America; 4 Microbiology Group, School of Biological Sciences, Illinois State University, Normal, Illinois, United States of America; 5 Department of Pediatrics, University of Chicago, Chicago, Illinois, United States of America; 6 Biomedical Informatics Research Center, Marshfield Clinic Research Foundation, Marshfield, Wisconsin, United States of America; 7 National Institute of Allergy and Infectious Diseases, Bethesda, Maryland, United States of America; Universidad Nacional de La Plata, Argentina

## Abstract

**Background:**

Linezolid (LZD) is beneficial to patients with MRSA pneumonia, but whether and how LZD influences global host lung immune responses at the mRNA level during MRSA-mediated pneumonia is still unknown.

**Methods:**

A lethal mouse model of MRSA pneumonia mediated by USA300 was employed to study the influence of LZD on survival, while the sublethal mouse model was used to examine the effect of LZD on bacterial clearance and lung gene expression during MRSA pneumonia. LZD (100mg/kg/day, IP) was given to C57Bl6 mice for three days. On Day 1 and Day 3 post infection, bronchoalveolar lavage fluid (BALF) protein concentration and levels of cytokines including IL6, TNFα, IL1β, Interferon-γ and IL17 were measured. In the sublethal model, left lungs were used to determine bacterial clearance and right lungs for whole-genome transcriptional profiling of lung immune responses.

**Results:**

LZD therapy significantly improved survival and bacterial clearance. It also significantly decreased BALF protein concentration and levels of cytokines including IL6, IL1β, Interferon-γ and IL17. No significant gene expression changes in the mouse lungs were associated with LZD therapy.

**Conclusion:**

LZD is beneficial to MRSA pneumonia, but it does not modulate host lung immune responses at the transcriptional level.

## Introduction

Community associated methicillin-resistant *Staphylococcus aureus* (CA-MRSA) is an emerging threat to human health throughout the world. Its epidemic spread and high mortality in healthy individuals have raised alarm in the biomedical community. In the USA, CA-MRSA infections are almost entirely attributed to a pandemic and highly virulent strain, USA300 [1,2]. CA-MRSA is the most predominant isolates in patients with noscomial pneumonia [3]. CA-MRSA pneumonia is characterized by a dramatic inflammatory response in the lungs, resulting in airway neutrophil influx, loss of alveolar structure, severe pulmonary edema, hemorrhage, intrapulmonary bacterial proliferation and high mortality rates [4,5].

Currently the American Thoracic Society and the Infectious Diseases Society of the America recommend vancomycin (VAN) or linezolid (LZD) as first-line agents for treatment of MRSA pneumonia [6,7]. Mechanistically, LZD selectively binds to the 50S ribosomal subunit, resulting in inhibition of bacterial protein synthesis [8]. Through this mechanism, LZD decreases *in vitro* production of exotoxins, including Panton Valentine Leukocidin (PVL), hemolysins, and enterotoxins. In addition, exotoxin release leads to a corresponding increase in induction of tumor necrosis factor, which can be also suppressed with LZD [9-11]. Using a murine model of pneumonia, Akinnusi et al. (2011) reported that LZD and VAN have comparable effects in modulating the expression of metalloproteinases (MMPs) in bronchoalveolar lavage fluid (BALF) and in regulating neutrophil activation [12]. They concluded that LZD exhibits a minimal modulatory effect on innate immunity in the animal model of MRSA pneumonia. All of these studies were conducted either via *in vitro* cell culture models or BALF characterization in a mouse model of MRSA pneumonia. Whether LZD modulates host lung immune responses at the mRNA level during MRSA pneumonia is still unknown. Previously, a rat necrotizing pneumonia model was used to study transcription of 84 genes mediating the early inflammatory response in the lung [5]. These genes include only inflammatory cytokines, chemokines and their receptors.

In this study, we hypothesized that LZD regulates host-bacteria interactions by modulating the gene expression profile in the lung during the MRSA pneumonia. Here we determined whether and how MRSA modulates host lung immune responses by genome-wide transcriptional profiling during pneumonia. In addition, we examined the corresponding effects of LZD on survival, lung bacterial clearance and acute lung injury.

## Materials and Methods

### Bacterial Preparation for Inoculation

USA300 CA-MRSA wild-type LAC strain [13,14] was used in this study. To prepare an inoculum for animals, a frozen stock of *S. aureus* was incubated onto a tryptic soy agar plate at 37°C overnight. A single colony was put into 3 ml tryptic soy broth (TSB) and incubated overnight in a shaker set at 250 rpm and 37°C. One milliliter of overnight culture was then grown in 100 ml TSB solution until OD_660_ was approximately 0.5. Fifty milliliter of bacteria were then centrifuged at 6000 g for 15 min at 4°C, washed in sterilized phosphate-buffered solution (PBS) and resuspended in a certain volume of PBS solution. In the sublethal model for bacterial clearance and gene expression profiling assays, the volume of PBS for bacterial resuspension is 1.5 ml, resulting in an estimated concentration of 1x10^8^ CFU per 30 µl volume. In the lethal model, the PBS volume was 750 µl, resulting in an estimated concentration of 3~4 x10^8^ CFU per 30 µl volume. After bacterial resuspension, the solution was immediately used for animal inoculation. All inocula were then quantified by plating serial dilutions in PBS on tryptic soy agar and counting colonies after overnight incubation at 37°C. In this study, the lethal model was used to study how LZD is beneficial to survival of mice after MRSA pneumonia. Since very few mice survive > 48 hrs in the lethal model without LZD therapy [13], the sublethal model was used for bacterial clearance and gene expression profiling assays.

### Animals and Procedures

All animal experiments and procedures were approved by the Institutional Animal Care and Use Committee in Northwestern University. The lung infection procedure described previously [13,14] was followed. Briefly, C57Bl6 male mice (7 weeks old, Charles River) were anesthetized before inoculation of 30 µl of USA300 CA-MRSA strain LAC suspension via the left nare. Animals were held upright for one minute post-inoculation and then placed into a cage in supine position for recovery. All animals were provided with food and water *ad libitum*. A small number of animal that succumbed within the first six hours post infection (< 1%) were excluded in the subsequent data analysis.

Mice were divided into two groups: placebo (PBS) and LAC. Each group included two subgroups: no LZD treatment and LZD treatment. Mice were treated with PBS or LZD starting at 6 hours post inoculation, prior to the peak expression of proinflammatory cytokines or chemokines. LZD was administered by intraperitoneal injection (I.P), 100 mg/kg/day for three days. The LZD concentration and treatment time selected in our model were based on previous studies [15-17] in order to achieve success against MRSA with minimum inhibitory concentration (MIC) as high as 2 to 4 μg/ml.

In our established mouse model of MRSA pneumonia, severely ill mice that show signs of ruffled fur, markedly decreased activity, surface temperature < 23°C, diminished righting reflex and decreased respiratory rate are euthanized. The mice are observed continuously within the first hour after MRSA inoculation, and then checked once per six hours for up to three days. In the lethal model, all the surviving mice after one week were euthanized.

### Lung Bacterial Clearance Assay

In the sublethal model, lungs were harvested after the mice were euthanized and homogenized in 1 ml sterilized PBS. Serial dilutions (1:10, 1:100) were prepared in PBS and plated on tryptic soy agar plates. After 24-hr incubation of the plates, bacterial colonies were counted and the number of colony forming units (CFU) per ml lung homogenate was calculated.

### Bronchoalveolar Lavage Fluid (BALF) Protein Concentration and Cytokine Measurement

BALF was performed by instilling 1 ml of cold Hank’s Salt solution (HBSS, Invitrogen, Grand Island, NY) via tracheal cannula, as previously described [18]. After centrifuge at 600 g for 10 min, the supernatant was harvested for protein concentration measurement by Pierce^®^ BCA protein assay kit (Thermo Scientific Inc, Rockford, IL).

The Bio-plexPro^TM^ mouse cytokine TH17 panel A kit (M60-00007NY, Bio-Rad Inc) was used to measure IL1β, IL-6, IL-17A, IFN-γ and TNF-α levels in BALF with 6 replications per group.

### Histology Preparation

A 20-gauge angiocath was sutured into the mouse trachea. The lungs were inflated with 0.8 ml 4% paraformaldehyde (PFA) and fixed in a 15 ml tube with the same concentration of PFA solution overnight at 4°C. The fixed lungs were paraffinized and 5-μm sections were stained with hematoxylin/eosin.

### Microarray Data and Functional Analysis

RNA expression analysis was performed by the Illumina MouseRef-8 BeadChip (Illumina, San Diego, CA), which provides coverage of approximately 25,700 genes and expressed sequence tags. Four independent mouse lung tissue samples at Day 1 and Day 3 post MRSA infection were randomly selected from each group and used for RNA isolation with RNeasy plus mini kit (Qiagen, Valencia, CA). RNA quality was checked by Agilent Bioanalyzer (Santa Clara, CA) and labeled by a commercial kit (TargetAmp 1-Round Aminoallyl-aRNA Ki; Epicentre, Madison, WI, USA). The labeled RNA was then hybridized to the Illumina MouseRef-8 BeadChip. Raw signal intensities of each probe were obtained by data analysis software (Beadstudio; Illumina) and imported to the Lumi package of Bioconductor for data transformation and normalization [19-21]. “Absent/Present” call detection was performed based on detected p value (p < 0.01). 15,490 out of 25,697 probes were considered valid signals.

Differentially expressed genes were identified by means of an Analysis of Variance (ANOVA) model with empirical Bayesian variance estimation [22]. The false discovery rate (FDR) was adopted to reduce false positives in multiple comparisons. Stringent criteria (fold change ≥ 1.5 up or down, *P* < 0.01, FDR < 0.05) were used to filter significantly differentially expressed genes. Two-dimensional hierarchical clustering was applied to these filtered probes to generate a global overview of the gene expression map (heat map).

Gene Ontology enrichment analysis of the significantly differentially expressed genes was performed by GeneAnswers, a Bioconductorpackage, based on hypergeometric test [23-25]. Microarray data was deposited in the Gene Expression Omnibus database with accession number GSE36587 and GSE46071 (GEO database, http://www.ncbi.nlm.nih.gov/geo/).

### Reverse Transcription and Real-time PCR Validation

Two microgram of purified RNA was reverse transcribed to single-strand cDNA using Taqman RNA reverse transcription kit (cat. #N8080234, Applied Biosystems Inc [ABI]). Real-time PCR was performed on an ABI 7900HT machine. Specific Taqman quantitative real-time PCR assays were ordered from ABI (specific assay IDs available upon request). The relative mRNA expression levels were normalized to the expression of a housekeeping gene, hexose-6-phosphate dehydrogenase (G_6_PDH), and determined by calculating the ΔΔCt value, as detailed in the manufacturer’s guidelines.

### Statistical Analysis

P-values for survival data were calculated using the Gehan-Breslow-Wilcoxon test, with a Wilcoxon rank-sum test for bacterial clearance data analysis. For analysis of BALF protein concentration and real-time PCR data, two-tailed *Student t-tests* were used for comparisons between two groups. One way analysis of variance (ANOVA) and pairwise *t*-test with Bonferroni-adjustment were used for comparison of over two groups. Results are displayed with mean ± SEM (standard error of the mean) for at least three independent experiments. P < 0.05 was considered statistically significant. All statistical analysis was performed on GraphPad Prism 5.1 (GraphPad Software, La Jolla, CA).

## Results

### (1) LZD Significantly Improves Survival and Lung Bacterial Clearance in MRSA-mediated Pneumonia

**Figure 1:**
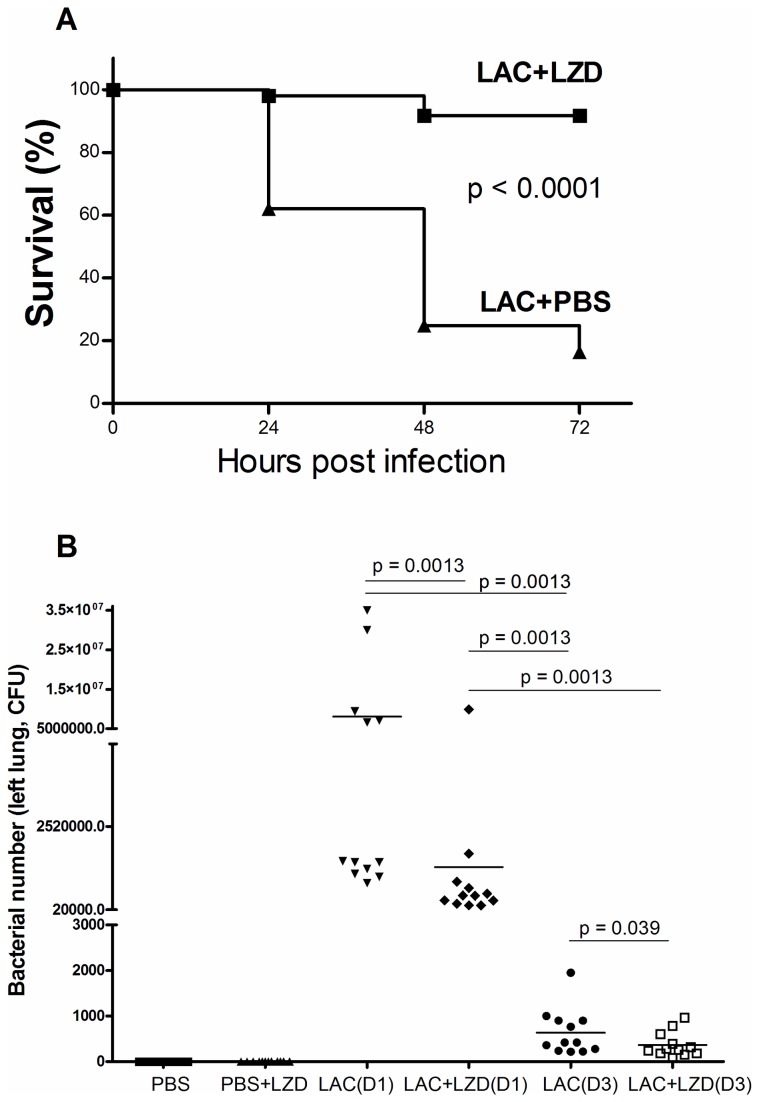
Survival and lung bacterial clearance data with Linezolid and no Linezolid therapy in MRSA mediated pneumonia. **A)** Survival data after 24 hrs, 48 hrs and 72 hrs post MRSA lung infections in a mouse lethal model of MRSA pneumonia (38 mice were used in LAC+LZD group and 39 mice in LAC+PBS group). p < 0.0001 calculated by a Gehan-Breslow-Wilcoxon test). **B)** Bacterial clearance data at Day 1 and Day 3 post MRSA infection in a mouse sublethal model of MRSA pneumonia. Twelve mice from three experiments were used in each group. P-values were calculated by a Wilcoxon rank-sum test.

In our lethal model, without LZD treatment, survival rates of LAC group at 24, 48 and 72 hrs post infection were 62.0 ± 9.0%, 24.8 ± 7.9% and 16.4 ± 2.0%, respectively. With LZD therapy, survival rates of LAC group at 24, 48 and 72 hrs post infection were 98.0 ± 2.0%, 91.7 ± 1.2% and 91.7 ± 1.2%, respectively (Figure 1A). All mice in the PBS or PBS+LZD groups survived. This clearly demonstrated that LZD significantly improved survival in the lethal mouse model of MRSA-mediated pneumonia (p < 0.0001).

In the sublethal mouse model, no deaths occurred in any groups (data not shown). We checked bacterial number in left lungs (right lungs were used for gene expression assay) at Day 1 and Day 3 post infection. The data were shown in Figure 1B. Comparing bacterial numbers between LZD and no LZD-treated group, LZD significantly improved bacterial clearance at both Day 1 post infection ([8.65 ± 3.68] x10^6^ CFU vs [1.38 ± 0.086] x10^6^ CFU, p = 0.0013), and at Day 3 post infection (1148 ± 509 CFU vs 287 ± 83 CFU, p = 0.039). In the group without LZD treatment, the bacterial burden in the lungs significantly decreased at Day 3 compared to Day 1 post MRSA infection (p = 0.0013).

### (2) LZD Significantly Attenuated MRSA-mediated Acute Lung Injury (ALI) Revealed by Lung Histology

**Figure 2:**
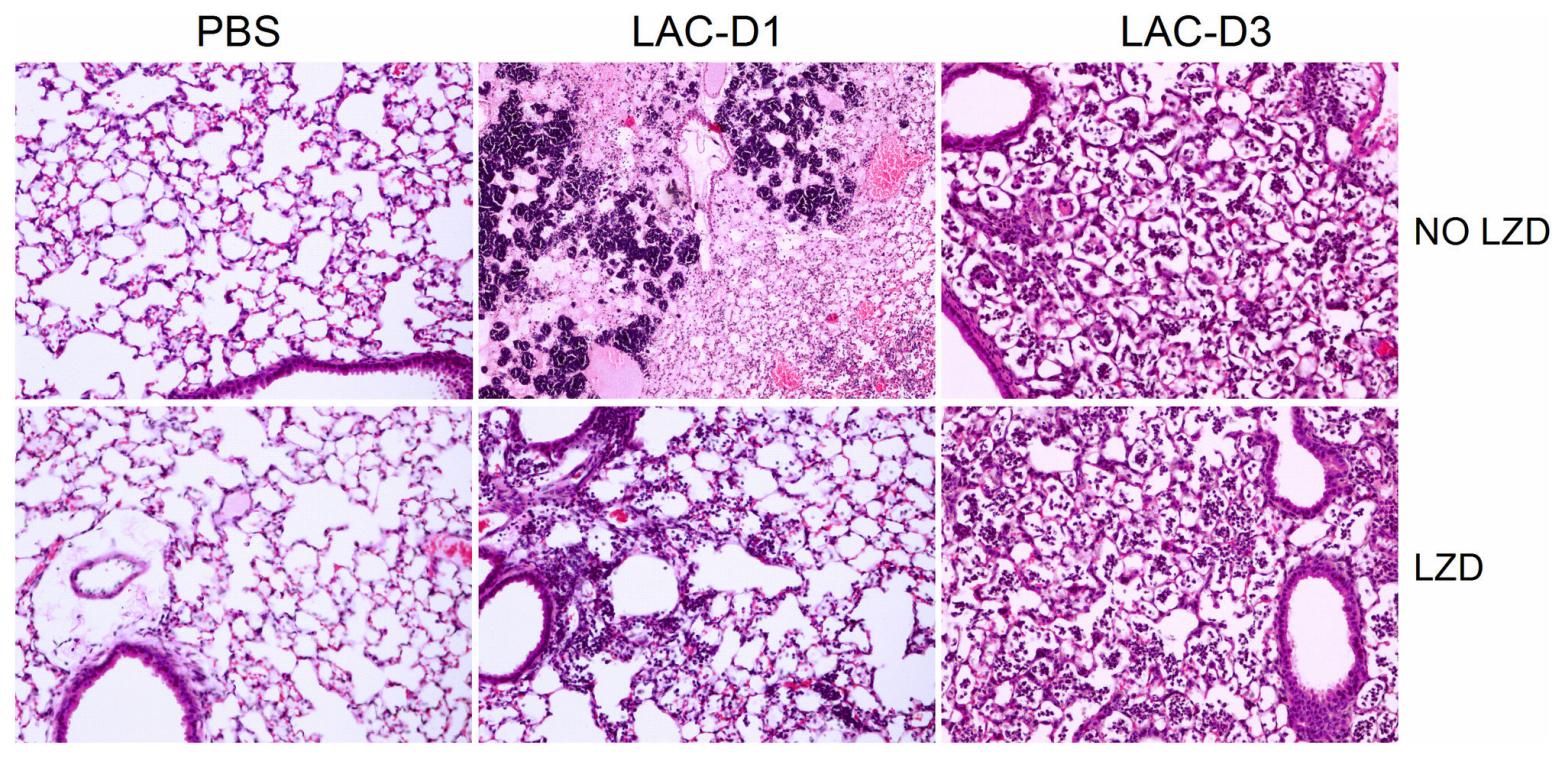
Lung hematoxylin and eosin (HE) staining images. The images in the first row are the representative groups without LZD therapy, and the images in the second row are the representative groups with LZD therapy. Magnification time, x20. Compared to the PBS control group, more inflammation with pulmonary edema, multifocal bacterial aggregates, and lung structure destruction was seen in the lungs at Day 1 post MRSA infection. With LZD treatment, no abscesses were formed, less lung edema and fewer inflammatory cells were observed compared to without LZD treatment. At Day 3 post MRSA infection, no multifocal bacterial aggregates could be found. However, the inflammatory infiltrates were still seen in the lung alveolar space.

Hematoxylin/eosin (HE) staining of lung tissues is shown in Figure 2. Compared to PBS group, more inflammation with pulmonary edema, multifocal bacterial aggregates, and lung structure destruction was seen in the lungs at Day 1 post MRSA infection. With LZD treatment, no abscesses formed, less lung edema occurred, and fewer inflammatory cells were observed compared to no LZD treatment. At Day 3 post MRSA infection, no multifocal bacterial aggregates could be found, although the inflammatory infiltrates were still seen in the lung alveolar space.

### (3) LZD Significantly Decreased BALF Protein Concentrations and Cytokine Levels during MRSA Pneumonia

**Figure 3:**
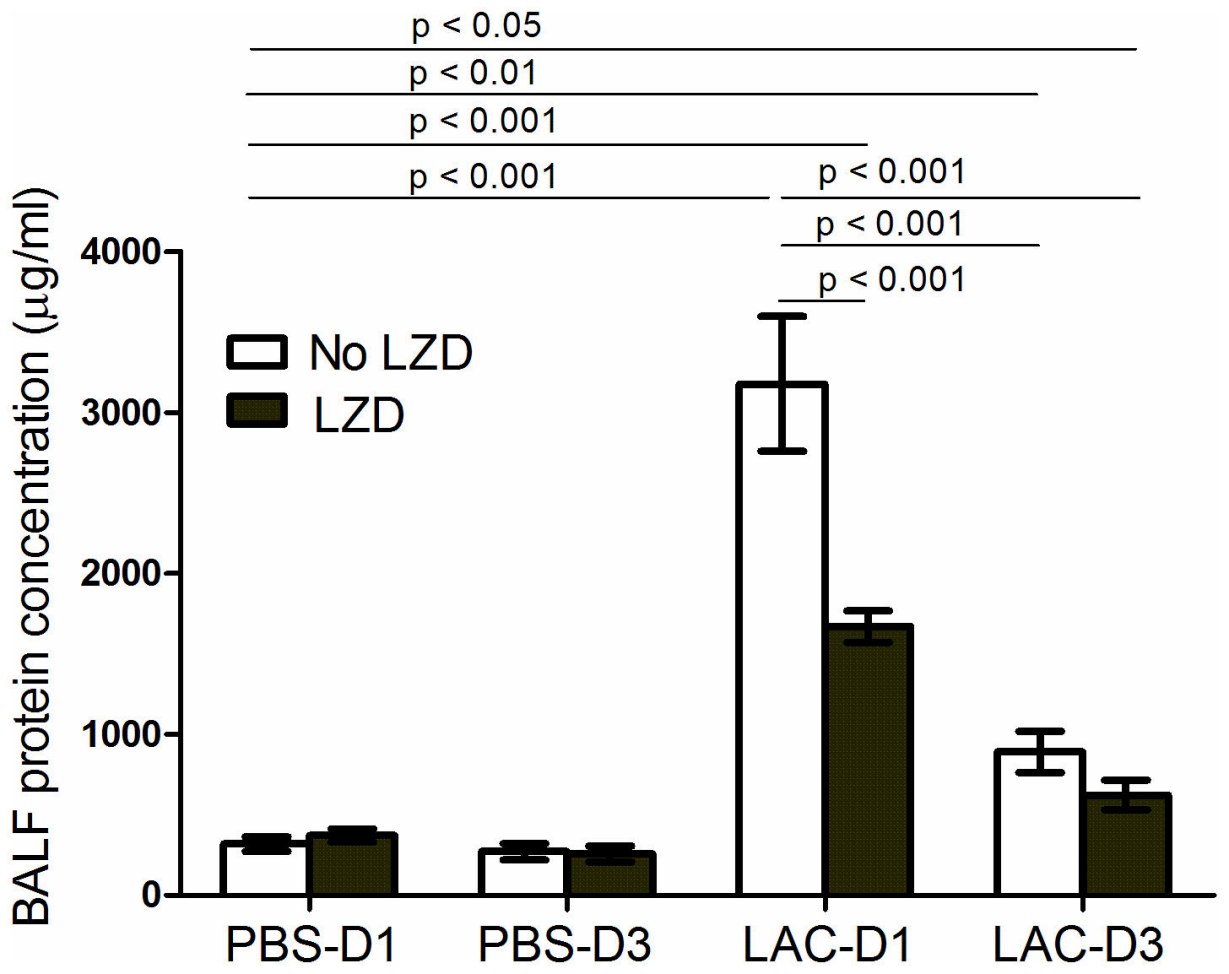
BALF protein concentration at Day 1 and Day 3 post MRSA lung infections. Values are the mean ± SEM (standard error of the mean) of three independent experiments (n = 12 in each group).

BALF protein concentrations dramatically increased in the LAC group, compared to the control group (PBS inoculated) (p < 0.001, Figure 3). LZD therapy significantly decreased BALF protein concentration in the LAC group at day 1 (3178.0 ± 418.4 µg/ml *vs* 1670.0 ± 98.8 µg/ml, p = 0.008) (Figure 3). It did not significantly change BALF protein concentration in control group (319.0 ± 46.2 *vs* 371.4 ± 41.8 µg/ml, p = 0.43). In the MRSA infected group, BALF protein concentration significantly decreased at Day 3 compared to Day 1 (3178.0 ± 418.4 µg/ml vs 890.3 ± 127.5 µg/ml, p < 0.001).

**Figure 4:**
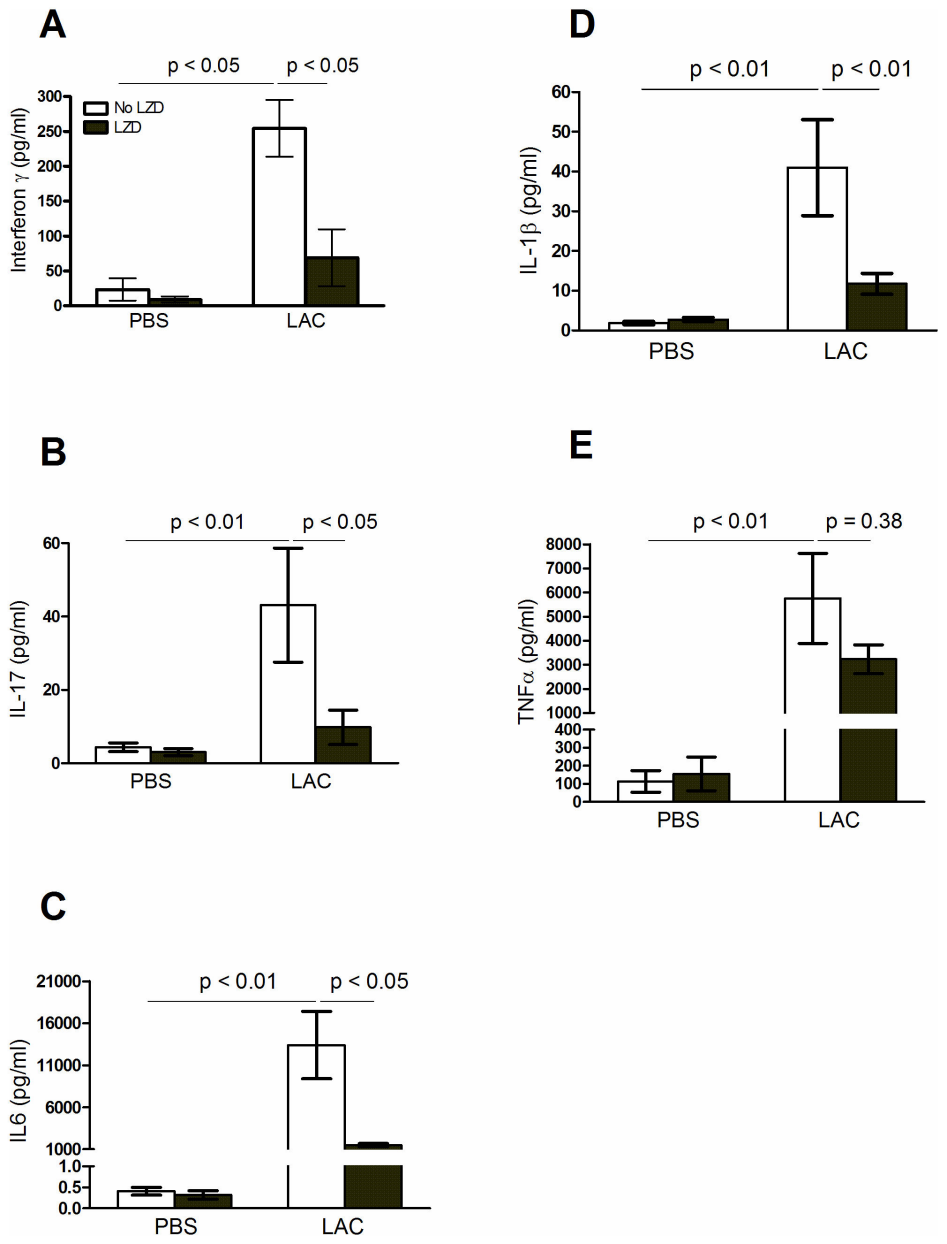
Interferon-γ, IL17, IL6, IL1β and TNFα levels in BALF samples at Day 1 post MRSA infections. Values are the mean ± SEM (standard error of the mean) of three independent experiments. There is no statistically significant difference in TNFα levels between the LAC and LAC+LZD groups (p = 0.38).

Our cytokine data (Figure 4) showed LZD significantly decreases IL1β, IL6, Interferon-γ and IL17 in LAC+LZD group, compared to LAC group. No significant difference was demonstrated for any cytokine measured between the PBS and PBS+LZD groups. In addition, no statistically significant difference in TNFα levels between the LAC and LAC+LZD groups (p = 0.38) was demonstrated.

### (4) Genome-wide Lung Gene Expression Profiles during MRSA Pneumonia

**Figure 5:**
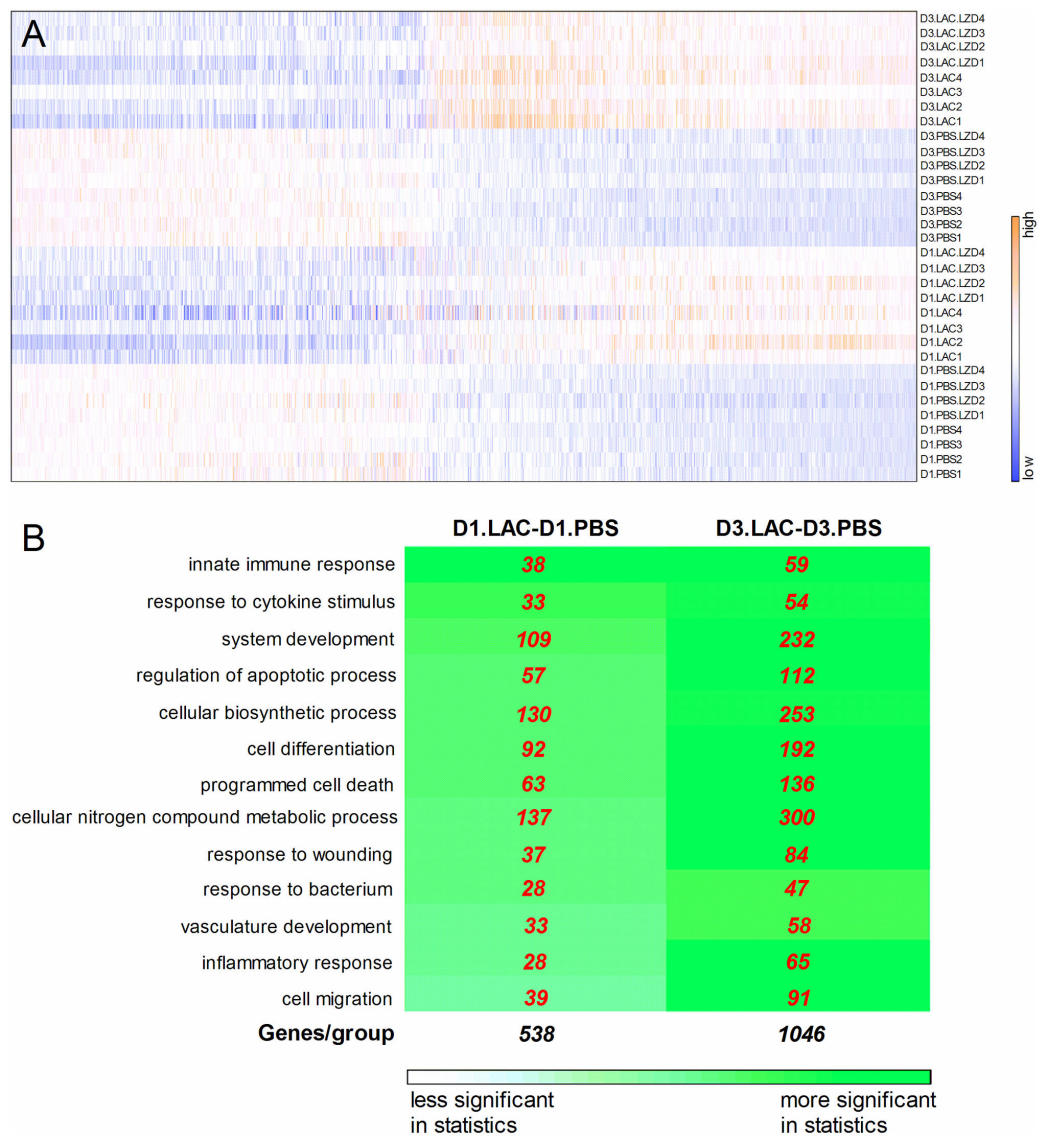
Genome-wide gene expression profiles in lungs at Day 1 and Day 3 post MRSA infection. **A**) Heat map of eight groups, with four replications per group. Each row represents one sample, and each column represents one gene. Fold change ≥ 1.5 up or down, *P* < 0.01, FDR < 0.05); **B**) Functional group concept-gene analysis for the comparison between LAC (Day 1) and PBS (Day 1) and between LAC (Day 3) and PBS (Day 3). The figure shows how many genes are involved in selected relevant biological pathways and which functions are significantly up-regulated or down-regulated between the LAC and PBS groups at Day 1 or Day 3 post MRSA infection. The background color for each cell is proportional to the hypergeometric test p value. The more saturated the color is, the smaller the p-value.

Genome-wide lung gene expression profiles of the four groups of mice (PBS, PBS+LZD, LAC and LAC+LZD) at Day 1 and Day 3 post MRSA lung infections were first examined and the heatmap is demonstrated in Figure 5A. Four biological replicates in each group showed highly consistent patterns.

A remarkable difference was demonstrated (Figure 5A) between PBS and LAC groups at either Day 1 or Day 3 post MRSA infection. Compared to PBS group, 538 and 1046 genes were significantly modulated (fold change ≥ 1.5 or ≤ -1.5) in lungs at Day 1 and Day 3 after MRSA infection, respectively. Of the 538 differentially expressed genes at Day 1, 261 (48.5%) were up-regulated and 277 (51.5%) were down-regulated; of the 1046 differentially expressed genes at Day 3, 602 (57.6%) were up-regulated and 444 (42.4%) were down-regulated. Table 1 lists 33 transcripts, selected according to their fold changes and functions, up-regulated or down-regulated at Day 1 or Day 3 post MRSA infection, compared to PBS group. All the up-regulated and down-regulated genes were listed in the Tables S1 and S2, respectively. Assignment of the significantly altered genes into the biological-process GO category (Figure 5B) revealed genes controlling innate immune response, inflammatory response, response to wounding, bacterium and cytokine stimulus, cell differentiation and migration, apoptosis, cellular nitrogen compound metabolic process, system development and vasculature development are significantly modulated by MRSA infection. In each biological process, transcriptions of more genes were significantly modified in the lungs at Day 3, compared to Day 1.

**Table 1:** Genes showing significantly different expression levels at Day 1 and Day 3 after MRSA lung infections as compared to a PBS control group.

Symbol	Description	Fold Change (DAY 1)	Fold Change (DAY 3)	Entrez ID
**Inflammatory response**				
Ccl3	chemokine (C-C motif) ligand 3	25.12	6.11	20302
Ccl4	chemokine (C-C motif) ligand 4	37.6	11.45	20303
Ccl7	chemokine (C-C motif) ligand 7	10.5	4.07	20306
Cxcl9	chemokine (C-X-C motif) ligand 9	25.77	22.43	17329
Il1rn	interleukin 1 receptor antagonist	12.8	3.98	16181
Orm1	orosomucoid 1	29.03	5.72	18405
Saa3	serum amyloid A 3	28.67	55.2	20210
**Innate defense response**				
Serpina3f	serine (or cysteine) peptidase inhibitor, clade A, member 3f	11.42	5.62	20717
Serpina3g	serine (or cysteine) peptidase inhibitor, clade A, member 3g	6.69	7.1	20715
Ido1	indoleamine 2,3-dioxygenase 1	10.73	3.62	15930
Lcn2	lipocalin 2	8.13	7.54	16819
GDF15	growth differentiation factor 15	6.01	unavailable	23886
Socs3	suppressor of cytokine signaling 3	5.28	2.02	12702
**Response to cytokine and reactive oxygen species**				
Mmp9	matrix metallopeptidase 9	3.13	2.14	17395
Prdx5	peroxiredoxin 5	2.78	2.21	54683
Cyp2a5	cytochrome P450, family 2, subfamily a, polypeptide 5	-5.56	-7.25	13087
Ephx1	epoxide hydrolase 1, microsomal	-3.82	-2.78	13849
Pon1	paraoxonase 1	-3.81	-3.63	18979
**Response to wounding**				
F10	coagulation factor X	7.04	3.55	14058
**Regulation of apoptosis**				
Hmox1	heme oxygenase (decycling) 1	3.92	3.05	15368
Casp4	caspase 4	3.45	2.73	12363
Bid	BH3 interacting domain death agonist	1.99	1.93	12122
Bcl3	B cell leukemia/lymphoma 3	1.98	unavailable	12051
Sod2	superoxide dismutase 2, mitochondrial	2.36	unavailable	20656
Cat	Catalase	-2.44	-1.94	12359
Ednrb	endothelin receptor type B	-3.23	-1.92	13618
Nox4	NADPH oxidase 4	-2.48	unavailable	50490
Cadm1	cell adhesion molecule 1	-3.28	unavailable	54725
**System Development**				
Timp1	tissue inhibitor of metalloproteinase 1	13.66	5.48	21857
Junb	Jun-B oncogene	5.5	1.88	16477
Egr1	early growth response 1	2.38	unavailable	13653
ESM1	endothelial cell-specific molecule 1	-2.41	-3.37	71690
EPAS1	endothelial PAS domain protein 1	-2.34	-4.59	13819

The genes listed in this table have been selected according to the degree of expression difference (fold changes) and functions.

### (5) LZD Did Not Modulate Lung Gene Expression Profiling during MRSA Pneumonia

Using the stringent criteria mentioned above (fold change ≥ 1.5 up or down, *P* < 0.01, FDR < 0.05) to filter significantly differentially expressed genes, we did not identify any genes whose expression levels were significantly different between the PBS and PBS+LZD groups or between the LAC and LAC+LZD at Day 1 or Day 3 post MRSA infection (Figure 5A).

### (6)Validation of Gene Expression Changes by Real-time PCR

**Figure 6:**
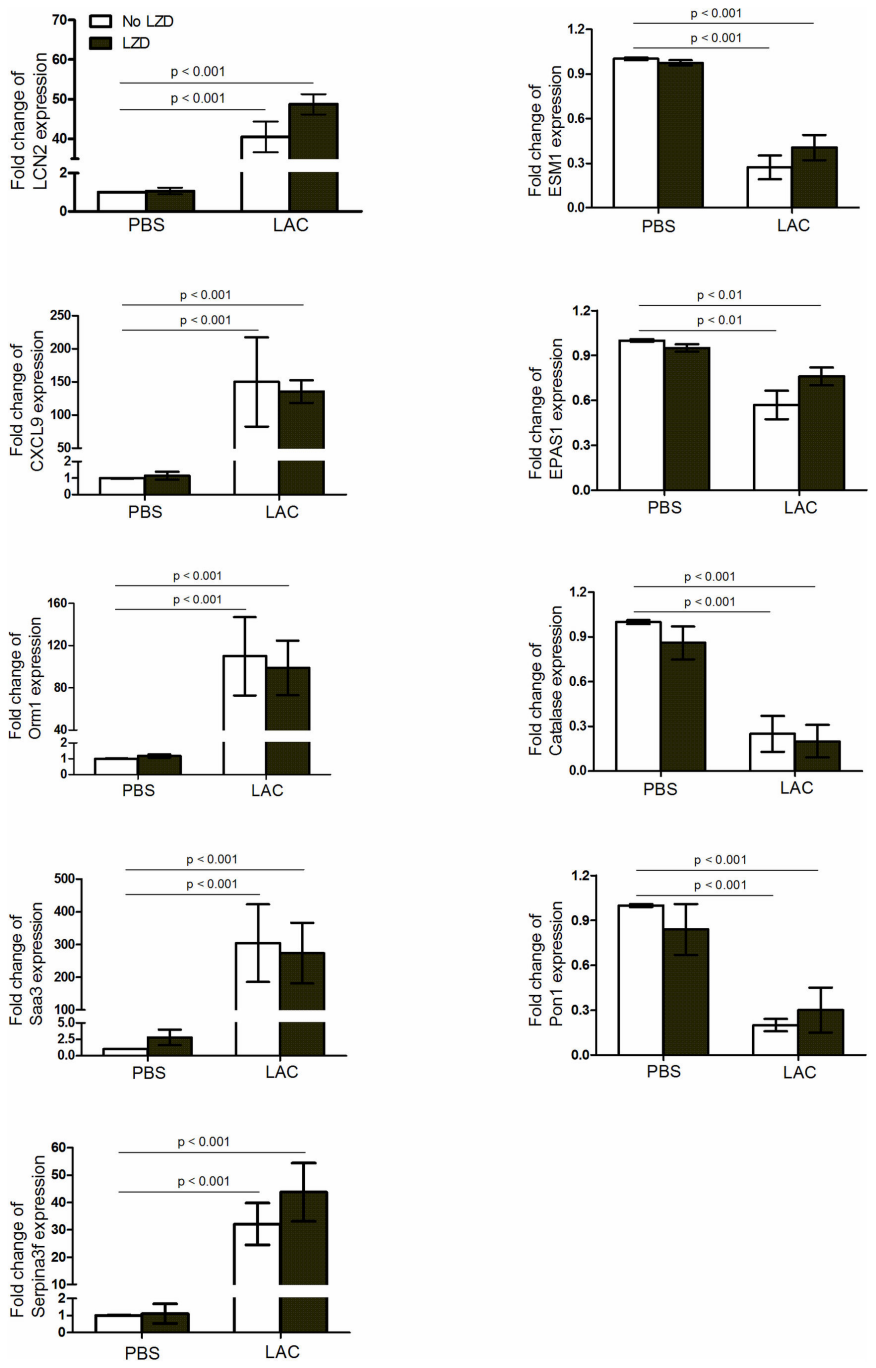
Quantitative real-time PCR confirmation of five up-regulated genes that were up-regulated during MRSA pneumonia (LCN2, CXCL9, Orm1, Saa3, Serpina3f) and four that were down-regulated (ESM1, EPAS1, Pon1, Catalase). There was no statistically significant difference between the PBS and PBS+LZD or between the LAC and LAC+LZD groups.

Nine genes were randomly selected from Table 1 for verification by real-time PCR using the Taqman gene expression assays. LCN2, SAA3, CXCL9, ORM1, Serpina3f were significantly up-regulated and EPAS1, ESM1, Pon1, Catalase were significantly down-regulated in lungs at Day 1 after MRSA infection, compared to the PBS group (Figure 6). No significant difference was seen between the LAC and LAC+LZD groups or between the PBS and PBS+LZD groups in all the genes tested (Figure 6). All real-time PCR data is consistent with cDNA microarray data.

## Discussion

In this study, we provide evidence showing that LZD improves survival in a mouse model of lethal MRSA pneumonia. Furthermore, in a corresponding sublethal model, LZD therapy was associated with significantly improved bacterial clearance, attenuated MRSA – mediated ALI, and decreased concentrations of BALF protein and cytokines such as IL1β, IL6, IFN-γ and IL17. However, these beneficial effects of LZD in MRSA pneumonia do not accompany with significant alterations in host lung transcriptional profile according to our microarray and real-time PCR data (Figures 5-6). This indicates that LZD may specifically target on modulating metabolism of MRSA and efficiently eliminate bacteria in lungs, but not exert adverse effects on the host lung tissues during MRSA pneumonia. This is the first study to show that LZD did not have an effect on genome-wide host lung innate immunity at the mRNA level. This novel finding is consistent with the previous findings reported by Akinnusi et al (2011), who showed that LZD exhibits a minimal modulatory effect on protein levels of IL-6 and MCP-5 in a murine model of MRSA-pneumonia [12]. However, Garcia-Roca et al (2006) demonstrated that LZD significantly suppressed the synthesis of the cytokines (IL6, TNFα, IL-1ra and IL-1β) in human peripheral blood mononuclear cells [26]. It is possible that LZD exerts different effects on cytokine production in *in vitro* cell culture models compared to animal models.

LZD decreased protein concentration and cytokine levels in BALF (Figures 3-4), which we attribute to greater bacterial clearance (Figure 1B), and thus lower cytokine levels were produced in the LZD-treated group compared to controls (Figure 4). In addition, the lung histological data showed that LZD significantly attenuated MRSA-mediated ALI (Figure 2). These findings further confirm that efficient elimination of MRSA from the lungs by LZD decreases the risk of pulmonary edema and acute lung injury.

To our knowledge, this study was the first to show the genome-wide lung gene expression profile changes at Day 1 and Day 3 post MRSA infection. Previously it was shown that inflammatory and blood coagulation components dominated the early response to MRSA in the lungs or airways [5,27]. Our microarray data not only confirmed inflammatory and blood components were significantly modulated by MRSA infection, but also revealed more transcriptional events during MRSA pneumonia. These events were listed in Figure 5B. Several major transcriptional events were highlighted here: 1) Inflammatory response was activated. Chemokine (C-C motif) ligand 3 (Ccl3), Ccl4, Ccl7, chemokine (C-X-C motif) ligand 9 (Cxcl9), Cxcl16, serum amyloid A3 (Saa3), orosomucoid 1 (Orm1) were up-regulated (Table 1). Ccl and Cxcl family members have chemoattractant activity and can elicit neutrophil recruitment to enhance bacterial clearance [28,29]; 2) Innate defense response was also activated. Serpina3, LCN2, Socs3, Ido1, GDF15 were significantly up-regulated. These proteins were reported to have an established or potential role in the innate defense against infections. For example, Ido1 has anti-microbial activity in a variety of infectious diseases [28], and LCN2 has been reported to be as a bacteriostatic protein for protection of airways against infection by *E. coli* [30]; 3) Components response to cytokines and reactive oxygen species were significantly modulated. These components included MMP9, Prdx5, Cyp2a5, Pon1 and Ephx1. They may play a protective role in regulation of cellular levels of cytokines and ROS, which are dramatically increased at the early stage of MRSA pneumonia. For example, Pon1 functions as an antioxidant [31] and Ephx1 plays an important role in the detoxification of electrophiles and oxidative stress [32]. Down-regulation of Ephx1 and Pon1 may ensure a certain amount of hydrogen peroxide, a potent antimicrobial agent, is present during pathogen infection; 4) Apoptosis-regulating proteins were significantly modulated. Hmox1, casp4, Bid, Bcl3, Sod2 were up-regulated and catalase, NOX4, Cadm1, Ednrb were down-regulated; 5) System development pathways were also significantly modified. Timp1, Junb, Egr1 were up-regulated and Esm1, Epas1 were down-regulated by MRSA infections. Esm1 and Epas1 have been shown to contribute to vascular angiogenesis [33-35]. Down-regulation of these components during MRSA pneumonia may dampen endothelium integrity. In general, endothelium integrity was considered as a “natural anticoagulant” [36]. Disruption of endothelium integrity can cause blood coagulation. This may explain why coagulation factor X (F10) was up-regulated during MRSA pneumonia.

There are a few limitations of this study. First, considering high mortality in the subgroup without LZD treatment (Figure 1A), only a sublethal mouse model of MRSA pneumonia (1 x 10^8^ CFU MRSA/mouse) was used to study the effect of LZD on host lung gene expression patterns. We have not tested whether gene expression patterns are affected by different MRSA inoculum. Second, we have tested the global host lung gene expression patterns only at Day 1 and Day 3 post MRSA infection. A substantial effect on host lung gene expression patterns during the first few hours after LZD therapy may therefore be missed. Third, the response of host cells may be affected by LZD in the same way as bacteria. LZD inhibits bacterial growth at the translational level and not at the transcriptional level [7]. It is possible that transcription in the host cells is not affected by LZD but translational levels are, similar to bacteria. Alternatively, LZD may have no effect on host innate immune cells. In this case, the benefit of LZD for MRSA pneumonia is solely greater antibiotic effect without immunomodulatory effects. In addition, it is possible that LZD affects transcriptional or translational events in the blood but not in the alveolar space. Therefore, in future studies, the use of lethal doses of MRSA given within the first few hours after LZD therapy and the analysis of protein levels in the lungs and blood are warranted.

In summary, our data show that LZD significantly improves survival, bacterial clearance and attenuates BALF protein concentration and cytokine levels. Lung cDNA microarray data show that LZD does not modulate lung gene expression profiles in our sublethal mouse model of MRSA pneumonia. To our knowledge, this study is the first report of genome-wide lung gene expression profiles during MRSA pneumonia. Description of global lung gene expression profiles is important to understand how murine lung host defense responds to MRSA pneumonia.

## Supporting Information

Table S1 All genes showing significantly different expression in lungs at Day 1 after MRSA lung infections as compared to a PBS control group. (XLS)

Table S2 All genes showing significantly different expression in lungs at Day 3 after MRSA lung infections as compared to a PBS control group. (XLS)
